# Moving from medical to health systems classifications of deaths: extending verbal autopsy to collect information on the circumstances of mortality

**DOI:** 10.1186/s41256-016-0002-y

**Published:** 2016-06-15

**Authors:** Lucia D’Ambruoso, Kathleen Kahn, Ryan G. Wagner, Rhian Twine, Barry Spies, Maria van der Merwe, F. Xavier Gómez-Olivé, Stephen Tollman, Peter Byass

**Affiliations:** 1 Institute of Applied Health Sciences, University of Aberdeen, Scotland, UK; 2 Umeå Centre for Global Health Research, Umeå University, Umeå, Sweden; 3 MRC/Wits Rural Public Health and Health Transitions Research Unit, School of Public Health, Faculty of Health Sciences, University of the Witwatersrand, Johannesburg, South Africa; 4 Directorate for Maternal, Child, Women and Youth Health and Nutrition, Mpumalanga Department of Health, Nelspruit, Mpumalanga South Africa; 5 INDEPTH: An International Network for the Demographic Evaluation of Populations and Their Health, Accra, Ghana

**Keywords:** Verbal autopsy, Social determinants, Health systems, Civil registration and vital statistics, Health surveillance, South Africa

## Abstract

**Background:**

Verbal autopsy (VA) is a health surveillance technique used in low and middle-income countries to establish medical causes of death (CODs) for people who die outside hospitals and/or without registration. By virtue of the deaths it investigates, VA is also an opportunity to examine social exclusion from access to health systems. The aims were to develop a system to collect and interpret information on social and health systems determinants of deaths investigated in VA.

**Methods:**

A short set of questions on care pathways, circumstances and events at and around the time of death were developed and integrated into the WHO 2012 short form VA (SF-VA). Data were subsequently analysed from two census rounds in the Agincourt Health and Socio-Demographic Surveillance Site (HDSS), South Africa in 2012 and 2013 where the SF-VA had been applied. InterVA and descriptive analysis were used to calculate cause-specific mortality fractions (CSMFs), and to examine responses to the new indicators and whether and how they varied by medical CODs and age/sex sub-groups.

**Results:**

One thousand two hundred forty-nine deaths were recorded in the Agincourt HDSS censuses in 2012–13 of which 1,196 (96 %) had complete VA data. Infectious and non-communicable conditions accounted for the majority of deaths (47 % and 39 % respectively) with smaller proportions attributed to external, neonatal and maternal causes (5 %, 2 % and 1 % respectively). 5 % of deaths were of indeterminable cause. The new indicators revealed multiple problems with access to care at the time of death: 39 % of deaths did not call for help, 36 % found care unaffordable overall, and 33 % did not go to a facility. These problems were reported consistently across age and sex sub-groups. Acute conditions and younger age groups had fewer problems with overall costs but more with not calling for help or going to a facility. An illustrative health systems interpretation suggests extending and promoting existing provisions for transport and financial access in this setting.

**Conclusions:**

Supplementing VA with questions on the circumstances of mortality provides complementary information to CSMFs relevant for health planning. Further contextualisation of the method and results are underway with health systems stakeholders to develop the interpretation sequence as part of a health policy and systems research approach.

**Electronic supplementary material:**

The online version of this article (doi:10.1186/s41256-016-0002-y) contains supplementary material, which is available to authorized users.

## Background

Despite increasing globalization, in many resource-poor countries, universal registration of vital events is lacking. In 2007, the Lancet Series *Who Counts* highlighted that the majority of births, deaths, and causes of deaths (CODs) in Africa and Asia are never recorded [46]. A further Lancet Series in 2015, *Counting Births and Deaths* estimated that 60 % of deaths worldwide pass without formal registration of medical cause [38]. The global deficit of information on the health of the world’s poor limits the capacity of the health system to respond, and raises fundamental questions about the links between material and data poverty [7].

Developing methods to record and analyse information on the deaths of people excluded from access to civil registration and vital statistics (CRVS) systems is therefore an important strategy for addressing health inequalities and saving lives. Verbal autopsy (VA) is a pragmatic approach in this regard used to determine levels and medical causes of death (CODs) for people who die outside health facilities and/or where the formal registration of deaths and medical causes is incomplete or absent. Applied in over 45 low and middle-income countries (LMICs) [2, 3, 52], VA is considered an effective means of population health data in lieu of functioning civil registration and vital statistics systems [38, 46].

A VA consists of two stages; firstly, trained fieldworkers interview final caregivers of the deceased (usually close relatives) according to a standard questionnaire on their medical signs and symptoms prior to death. In the second stage, the interview data are interpreted, until recently by physicians, to conclude probable medical causes. To date, VAs have mainly been conducted in research settings and/or as part of large household surveys that generate cause-specific mortality fractions (CSMFs) representative of disease burdens in populations.

In response to its widespread application, the WHO publishes standard VA instruments to harmonise international data collection and facilitate cross-national comparison and analysis [53–55, 57]. Acknowledging the global deficit in COD registration, the WHO released a short-form VA (SF-VA) in 2012 and advocated for VA in CRVS [48]. In 2014, the SF-VA was updated with a reiteration of this applicability [57]. Two Ministerial Summits in Africa and South Asia have since addressed the adoption of VA in CRVS. These shifts reflect the considerable momentum that has developed around the application of VA beyond a research method, as a scalable alternative for state CRVS systems [49, 50].

As VA transitions towards routine use, automated methods to interpret standardised VA data have also been developed. Probabilistic and algorithmic models that can process large volumes of data with 100 % internal validity and consistency [9] eliminate the need for separate physician interpretation, a stage that has been demonstrated to be timely, costly and inconsistent [8, 18, 42]. Most recently, VA has been adapted for use in smartphone applications [5]. This development opens further possibilities of scale and raises important considerations about whether and how to share COD conclusions with respondents at the time of interview.

‘Social Autopsies’ (SAs) are a further stream of methodological development. SAs seek to understand how and why deaths occur relative to particular social contexts [51]. SAs examine household, community and health systems determinants of deaths, such as knowledge, behaviours, accessibility and quality of care often using qualitative and mixed-methods approaches. The first comprehensive literature review of SA and the first standard SA instrument were published in 2011 [27, 28]. In 2016, VA and SA were used in an integrated format at national level [4, 29, 31]. SAs acknowledge the social determinants of particular forms of mortality and provide complementary information for service planning and resource allocation.

In this paper, we consider VA in terms of its development for routine application. To date, VA has been used mainly in research settings where survey findings are supplemented with additional information and analyses. When VA is applied routinely, these additional data and interpretations may not be available or possible in the same way. The overall purpose therefore relates to the WHO’s current efforts to develop a stand-alone tool that can be used routinely, including in situations where limited additional data are available [34].

The aims were to develop a system to a collect and interpret information on social and health systems determinants of deaths investigated in VA. This was based on the premise that deaths investigated in VA are likely to have occurred among people facing social exclusion from access to health systems. The objectives were to develop new VA indicators to capture information on background characterises of deaths (care processes, circumstances and events) related to the health systems and social contexts, and to explore how data yields could be interpreted. The work was primarily methodological, and sought to generate substantive information of practical relevance in the methodological development process.

## Methods

### Data collection

**Fig. 1:**
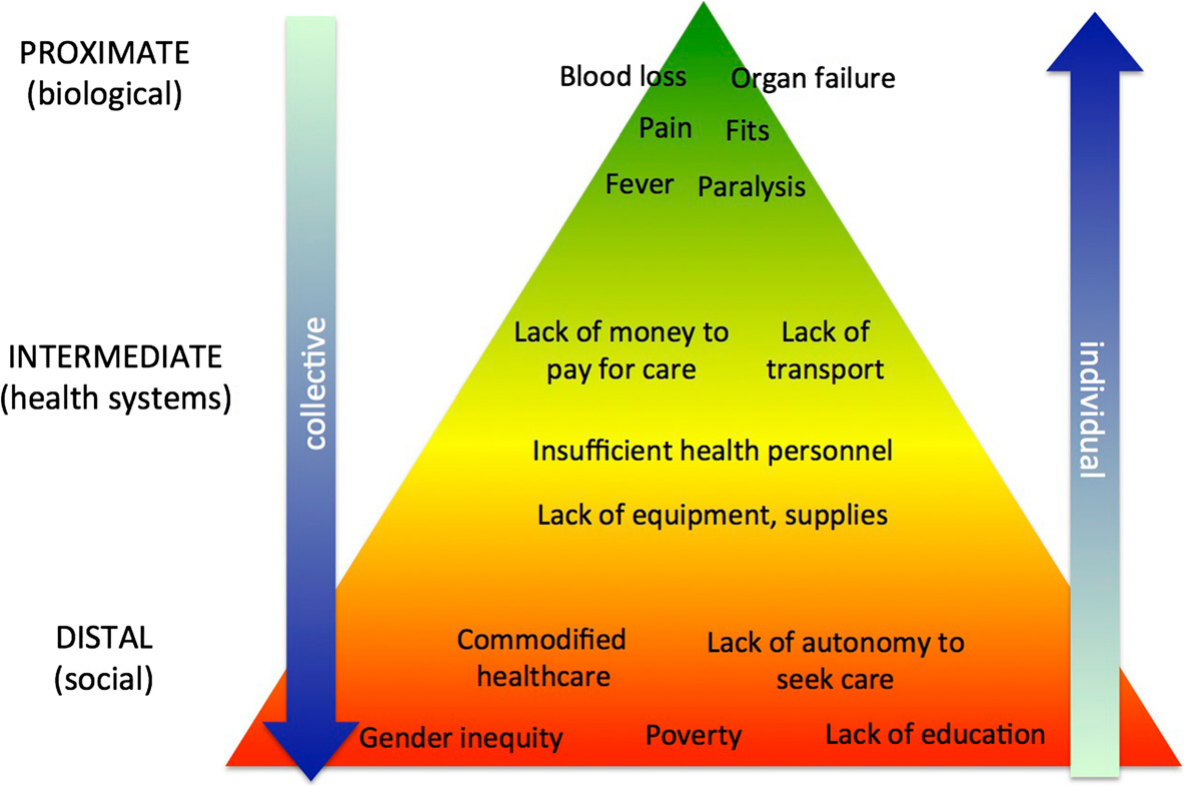
Conceptual framework of the determinants of health outcomes

As a first step towards identifying new indicators on the circumstances of mortality, we developed a conceptual model relating social and health systems factors to health outcomes. This was based on a classic model of child mortality that organises determinants of outcomes as proximate, intermediate and distal [40]. Proximate factors include biological processes and conditions that immediately precede outcomes. Intermediate and distal factors refer to the health systems, socio-economic and cultural conditions [40]. In Fig. 1, these categories (with examples) are arranged in a pyramid, with proximate determinants located at the apex and distal factors at the base to represent their individual to collective natures.

In this arrangement, intermediate factors, located at the interface of the two extremes, can be considered to have a mediating function between social conditions and health outcomes. This view is consistent with recent models of health systems as ‘core social institutions’, i.e. products of the relationships between patients and health workers, managers and policy makers etc., which as a whole establish social norms and values over eligibility for access to good quality care [17, 19, 20]. These models centralise a human and relational nature of the health system consistent with a people-centred health systems discourse [47] and have developed the debate from ‘hardware’ or ‘building blocks’ models of health systems in recent years [21, 43].

Given the theoretical ability to illuminate the relationships between social contexts and health outcomes, health system factors were adopted as the focus of the new indicators. Based again on the core social institutions models, it was assumed that health systems factors could be meaningfully represented in the care processes, circumstances, events and interactions of users and providers of services at and around the time of death. On this basis, literature on VA and SA from 2011 backwards was reviewed to identify relevant questions in other published VA tools on care interactions and processes at and around the time of death.

The 2007 WHO VA contains ten questions on contact with health services, places where care was received, contacts with health services, treatments provided, and health worker reported COD, with a similar format for child and infant deaths [54]. Kalter’s review of SA identifies recognition of severe illness, times and types of care sought, care seeking delays, and quality of care as relevant processes [28]. Kallander and colleagues’ standard SA also focuses on care interactions [27]. Pathways-to-care are examined in the health care utilisation module in 19 questions for adult deaths, 42 for child deaths and 61 for neonatal deaths on symptoms, care seeking, treatments, costs of care, transport and associated expenses (Additional file 1: Table S1).

Aspects of recognition of severity, access to and quality of care identified by Kalter and colleagues [28] were adopted as categories to which the new indicators were related. Drawing on Kallander’s instrument [27], questions on affordability were also included. Other than demographic and basic information on education, occupation and marital status, the 2007 WHO VA standard does not contain questions on social, economic and cultural (distal) factors [54]. Questions on contextual conditions were therefore configured to capture asset ownership, as it was relevant to the care seeking process. Considering these and additional studies and datasets [1, 22], ten indicators on key aspects of care seeking and utilisation at and around the time of death were constructed (Table 1).

**Table 1:** Questions on social and health systems factors at and around the time of death

	Theme	Question
↓ Care Pathway Home-To-Hospital ↓	Recognition of severity	In the final days before death, were there any doubts about whether medical care was needed?
		In the final days before death, was traditional medicine used?
	Mobilising assets to seek care	In the final days before death, did anyone use a telephone or cell phone to call for help?
		Did (s)he use motorised transport to get to the hospital or health facility?
	Access to care	Over the course of illness, did the total costs of care and treatment prohibit other household payments?
		In the final days before death, did s/he travel to a hospital or health facility?
		Does it take more than 2 h to get to the nearest hospital or health facility from the deceased's household?
	Quality of care	Were there any problems during admission to the hospital or health facility?
		Were there any problems with the way (s)he was treated (medical treatment, procedures, interpersonal attitudes, respect, dignity) in the hospital or health facility?
		Were there any problems getting medications, or diagnostic tests in the hospital or health facility?

### Study setting

**Fig. 2:**
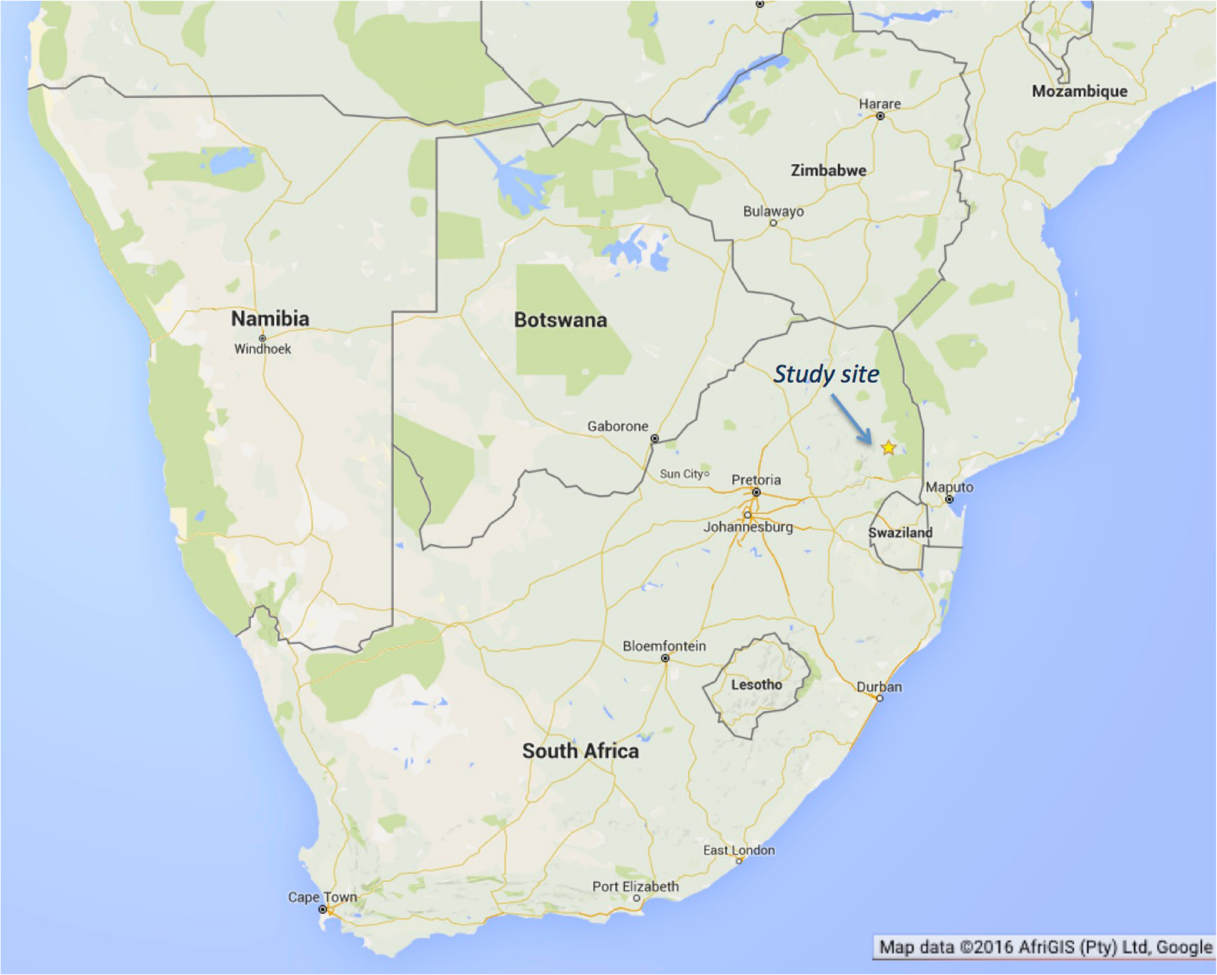
Map of South African Medical Research Council and the University of Witwatersrand’s Agincourt Health and Socio-Demographic Surveillance Site (HDSS) Bushbuckridge, Mpumalanga

The indicators were subsequently piloted in the South African Medical Research Council and the University of Witwatersrand’s Agincourt Health and Socio-Demographic Surveillance Site (HDSS) in the rural Bushbuckridge sub-district of Mpumalanga province in 2012 (Fig. 2). The Agincourt HDSS is a major research centre on population health established in 1992 in response to the absence of health information on rural populations in the country [25]. The site conducts annual censuses, collecting data on births, deaths and migrations in a population of approximately 110,000 occupying 21,000 households across 31 villages [26].

South Africa is a unique setting to develop systems to record information on social and health systems determinants of mortality. South Africa is simultaneously one of the strongest economies in the region yet one of the most unequal societies in the world [39]. Similarly, despite progressive and inclusive health policies [37], the health system is deeply divided with persistent disconnects between policy and implementation [11]. Collecting information on how health policy is ‘brought alive’ through care pathways and interactions as the processes of implementation was therefore thought to have the potential to provide useful information for remedial actions. Following a series of pilot interviews in Agincourt, the questions were refined for meaning and flow, and were subsequently integrated by the WHO into the short form (SF-VA) international standard in 2012 [12, 55]. Thereafter, the SF-VA standard was adopted in the Agincourt HDSS as part of the routine census activities.

### Data analysis

Following two annual census rounds in 2012 and 2013 in the Agincourt HDSS where the SF-VA had been applied, VA data were obtained for analysis. The VA data were analysed in two stages. Firstly, InterVA-4 was used to determine overall levels and medical causes of deaths from the indicators on medical signs and symptoms. InterVA-4 is a public-domain probabilistic model for VA data interpretation that computes VA input indicators as defined in WHO VA instruments and delivers CODs compatible with the International Classification of Diseases version 10 (ICD-10) [10]. CSMFs were generated and arranged in terms of magnitude and rank order, and according to age/sex sub-groups.

Secondly, responses to the new indicators were subject to descriptive analysis. The new indicators were grouped thematically according to a home-to-hospital care pathway sequence to aid interpretation (Table 1), with the questions on assets amalgamated into the access theme. All ‘yes’ responses were counted with the exceptions of the questions on assets and travelling to a hospital or health facility. For these questions, ‘no’ responses were counted to quantify reports of not travelling to a hospital, not using a cellphone to call for help, and not using motorised transport. Frequencies of responses were tabulated in absolute and relative terms, and according to medical COD and age/sex sub-groups. The frequencies were calculated as proportions of the number of deaths and for the indicators on the use of motorised transport and quality of care, the number of deaths that had travelled to a facility.

In both elements of the analysis, tests of significance were not required as the data were drawn from complete enumeration, and given the methodological nature of the work concerned with establishing internal validity and an interpretation sequence.

### Ethical considerations

The research was a secondary analysis of existing data collected via longitudinal surveillance for which ethical clearance was not required. The routine surveillance in Agincourt HDSS is approved by the Committee for Research on Human Subjects of the University of the Witwatersrand (protocol M960720, renewal approval number: M110138). Informed consent is obtained at individual and household levels, and community consent from traditional leaders, secured at the start of surveillance, is reaffirmed regularly.

## Results

### Medical causes of death

One thousand two hundred forty-nine deaths were recorded in the 2012/13 censuses, of which 1,196 (96 %) had complete VA data. For each death investigated there was one respondent who was interviewed using the Agincourt VA tool based on the WHO 2012 SF-VA [55]. According to InterVA analysis, the leading COD was acute respiratory infection including pneumonia, accounting for 14.4 % of the total burden. HIV/AIDS-related deaths and pulmonary TB accounted for 14.3 % and 12.9 % respectively. Cardiac diseases accounted for 7.2 % of deaths, with asthma and stroke accounting for 7.0 % and 5.6 %. 45 % of all deaths were among adults 15–49 years, and 10 % were under-5 years (Additional file 2: Table S2).

These six CODs accounted for 61.4 % of all deaths. According to InterVA4, further 41 specific CODs accounted for the remainder. As each of these CODs accounted for 5 % or less of the total burden, they were amalgamated into categories of COD to aid interpretation (Additional file 3: Table S3). According to this analysis, 47.0 % of deaths were the results of infectious diseases with 39.1 % attributed to due to non-communicable conditions. 7.2 % were due to external causes and 1.6 % and 0.6 % to neonatal and maternal conditions respectively. The causes of 4.5 % were indeterminable (Table 2).

**Table 2:** Cause category specific mortality fraction: all deaths, age and sex sub-groups

Category of COD	Neonate (<28 d)	Infant (1–11 m)	Under 5 (1–4 y)	Child (5–14 y)	Adult (15–49 y)	Mid-age (50–64 y)	Elder (65-84+ y)	Female	Male	Total number of deaths *n* (%)
Infectious		27 (4.8)	40 (7.1)	11 (2.0)	315 (56.0)	74 (13.2)	95 (16.9)	286 (50.9)	276 (49.1)	562 (47.0)
Non-communicable		2 (0.4)	5 (1.1)	1 (0.2)	129 (27.6)	82 (17.5)	249 (53.2)	269 (57.5)	199 (42.5)	468 (39.1)
External		2 (2.3)	6 (7.0)	3 (3.5)	60 (69.8)	11 (12.8)	4 (4.7)	16 (18.6)	70 (81.4)	86 (7.2)
Indeterminate	7 (13.0)		2 (3.7)	2 (3.7)	26 (48.1)	5 (9.3)	12 (22.2)	34 (63.0)	20 (37.0)	54 (4.5)
Neonatal	16 (84.2)	2 (10.5)	1 (5.3)					9 (47.4)	10 (52.6)	19 (1.6)
Maternal					7 (100.0)			7 (100.0)		7 (0.6)
Female	14 (60.9)	22 (66.7)	23 (42.6)	5 (29.4)	291 (54.2)	63 (36.6)	203 (56.4)			621 (51.9)
Male	9 (39.1)	11 (33.3)	31 (57.4)	12 (70.6)	246 (45.8)	109 (63.4)	157 (43.6)			575 (48.1)
Total number of deaths *n* (%)	23 (1.9)	33 (2.8)	54 (4.5)	17 (1.4)	537 (44.9)	172 (14.4)	360 (30.1)	621 (51.9)	575 (48.1)	1196 (100)

Disaggregating by age/sex sub-groups, higher levels of infectious mortality were observed in younger age groups whereas among deaths to people 50 years and over there were high proportions of non-communicable mortality. Otherwise, COD category-specific fractions were broadly similar in males and females with the exception of external (18.6 % versus 81.4 %), indeterminate (63.0 % versus 37.0 %) and maternal conditions (100 % versus 0 %) (females and males respectively) (Table 2).

### Social and health systems factors

**Fig. 3:**
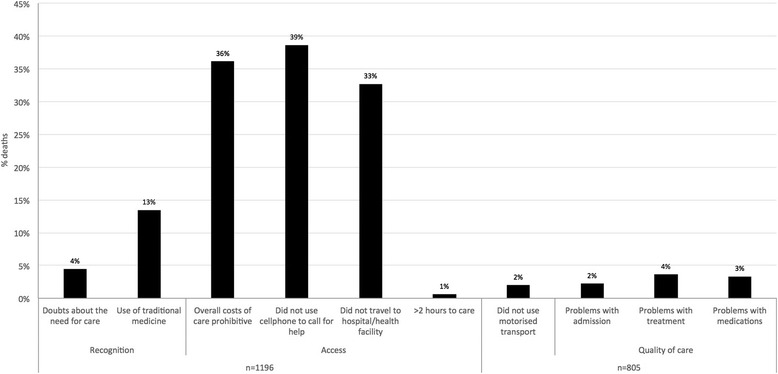
Frequencies of responses to new social and health systems indicators, all deaths (*n* = 1,196)

The majority of problems reported according to the new indicators related to access to services in the final days before death. In 38.6 % of all deaths, a cellphone had not been used to call for help, in 36.1 % costs of care were prohibitive and 32.7 % of the deceased did not travel to a hospital or health facility at the time of death. In terms of recognition of severity, 13.5 % of deaths had used of traditional medicine at the time of death, and 4.4 % had doubts about the need for care. Quality of care appeared to be less problematic. Of those who travelled to a facility (805/1196, 67.3 %), small proportions reported problems with the way they were treated (3.6 %), accessing medication (3.4 %) and admission (2.2 %). Only 2.0 % of those who travelled to a facility did so without motorised transport and 1.0 % of deaths had journeys of over two hours (Table 3, Fig. 3).

**Table 3:** Absolute and relative frequencies of social and health systems indicators as proportions of numbers of deaths, by COD categories

	Recognition		Access					Quality of care			
Category of COD	Doubts about the need for care	Use of traditional medicine	Overall costs prohibitive	Did not use cellphone	Did not travel to hospital/ health facility	>2 h to hospital/ health facility	Did not use motor transport^a^	Problems with admission^a^	Problems with treatment^a^	Problems with medications^a^	Total number of deaths *n* (%)
Infectious	28 (5.0)	102 (18.1)	254 (45.2)	178 (31.7)	123 (21.9)	6 (1.1)	9 (2.1)	8 (1.8)	17 (3.9)	19 (4.3)	562 (47.0)
Non-communicable	22 (4.7)	53 (11.3)	165 (35.3)	168 (35.9)	154 (32.9)	1 (0.2)	6 (1.9)	8 (2.5)	10 (3.2)	8 (2.5)	468 (39.1)
External		1 (1.2)	4 (4.7)	71 (82.6)	71 (82.6)		1 (6.7)				86 (7.2)
Indeterminate	3 (5.6)	4 (7.4)	6 (11.1)	31 (57.4)	34 (63.0)			2 (10.0)	1 (5.0)		54 (4.5)
Neonatal		1 (5.3)	3 (15.8)	11 (57.9)	7 (36.8)						19 (1.6)
Maternal				3 (42.9)	2 (28.6)				1 (20.0)		7 (0.6)
											1196 (100.0)
Total number of indicators reported n (%)	53 (4.4)	161 (13.5)	432 (36.1)	462 (38.6)	391 (32.7)	7 (0.6)	16 (2.0)	18 (2.2)	29 (3.6)	27 (3.4)	

^a^Denominator for the relative frequency was the number of deaths that had travelled to a hospital or health facility

N.B. Respondents were able to indicate more than one social and health system indicator for each death reported. Proportional frequencies of the new indicators therefore sum to >100 %

**Fig. 4:**
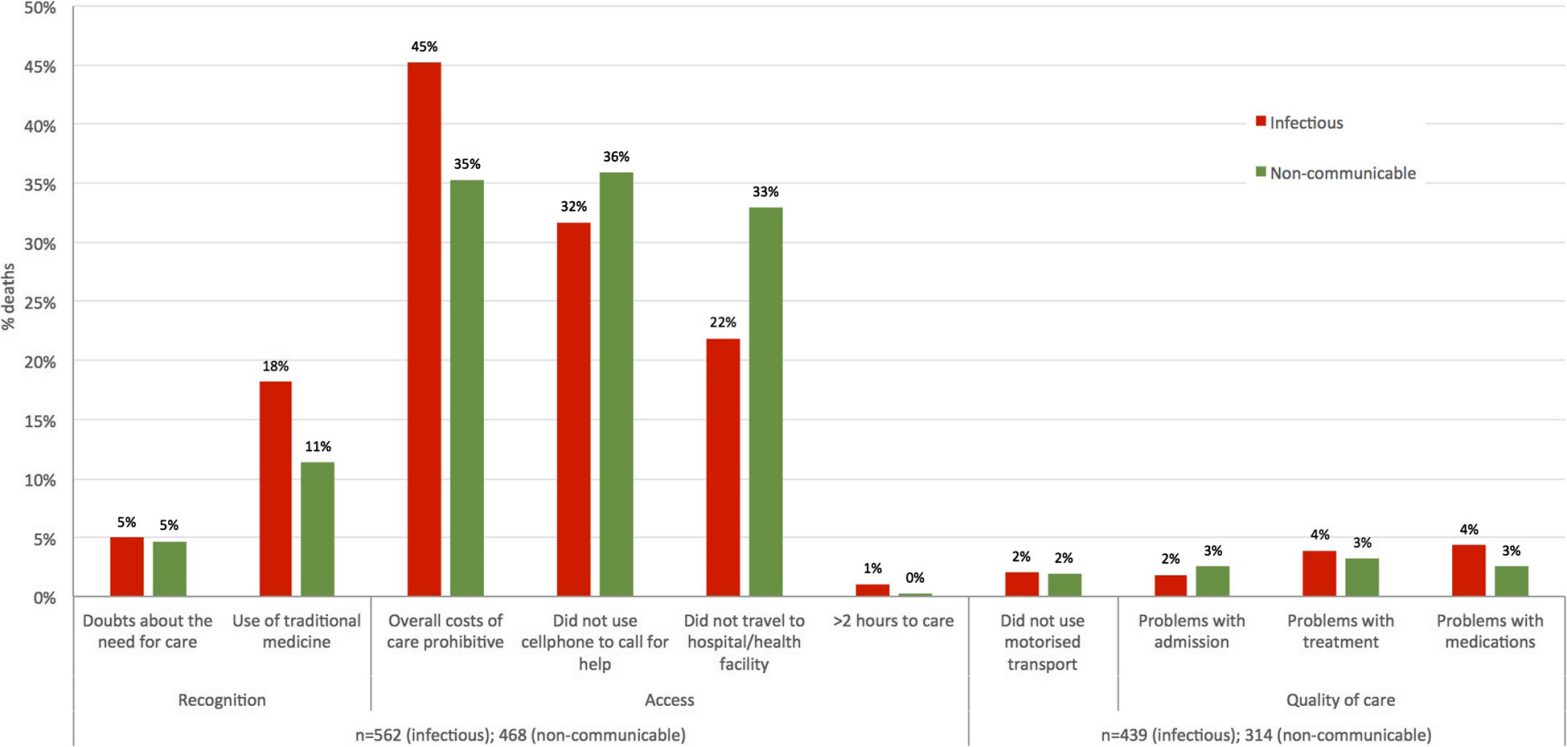
Frequencies of responses to new social and health systems indicators, infectious and non-communicable deaths (*n* = 562 and 468)

This pattern was consistent across age, sex and COD category sub-groups. For infectious and non-communicable deaths (*n* = 1,030), 45.2 % and 35.3 % had found care unaffordable. In 31.7 % of the infectious deaths and 35.9 % of deaths due to NCDs, a cellphone had not been used to call for help. In addition, 21.9 % of infectious disease deaths and 32.9 % of deaths due to non-communicable conditions had not travelled to a hospital or facility at the time of death. Use of traditional medicine at the time of death was also consistent with the overall trend (18 % and 13 % for deaths owing to infectious and NCDs respectively). The remaining indicators were reported in 5 % or less of deaths from infectious and NCDs (Fig. 4) (Additional file 4: Table S4).

**Fig. 5:**
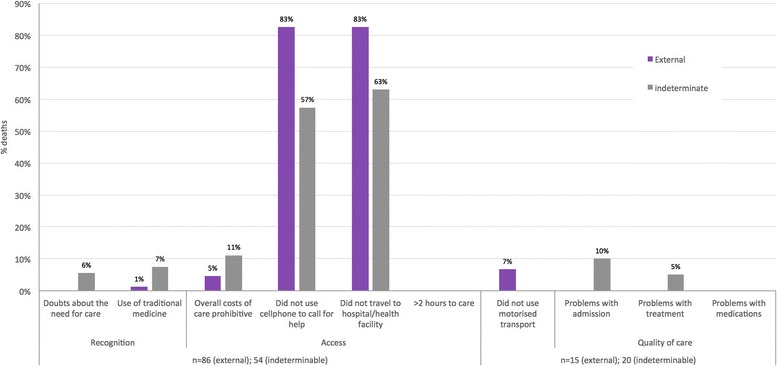
Frequencies of responses to new social and health systems indicators, external and indeterminate deaths (*n* = 86 and 54)

**Fig. 6:**
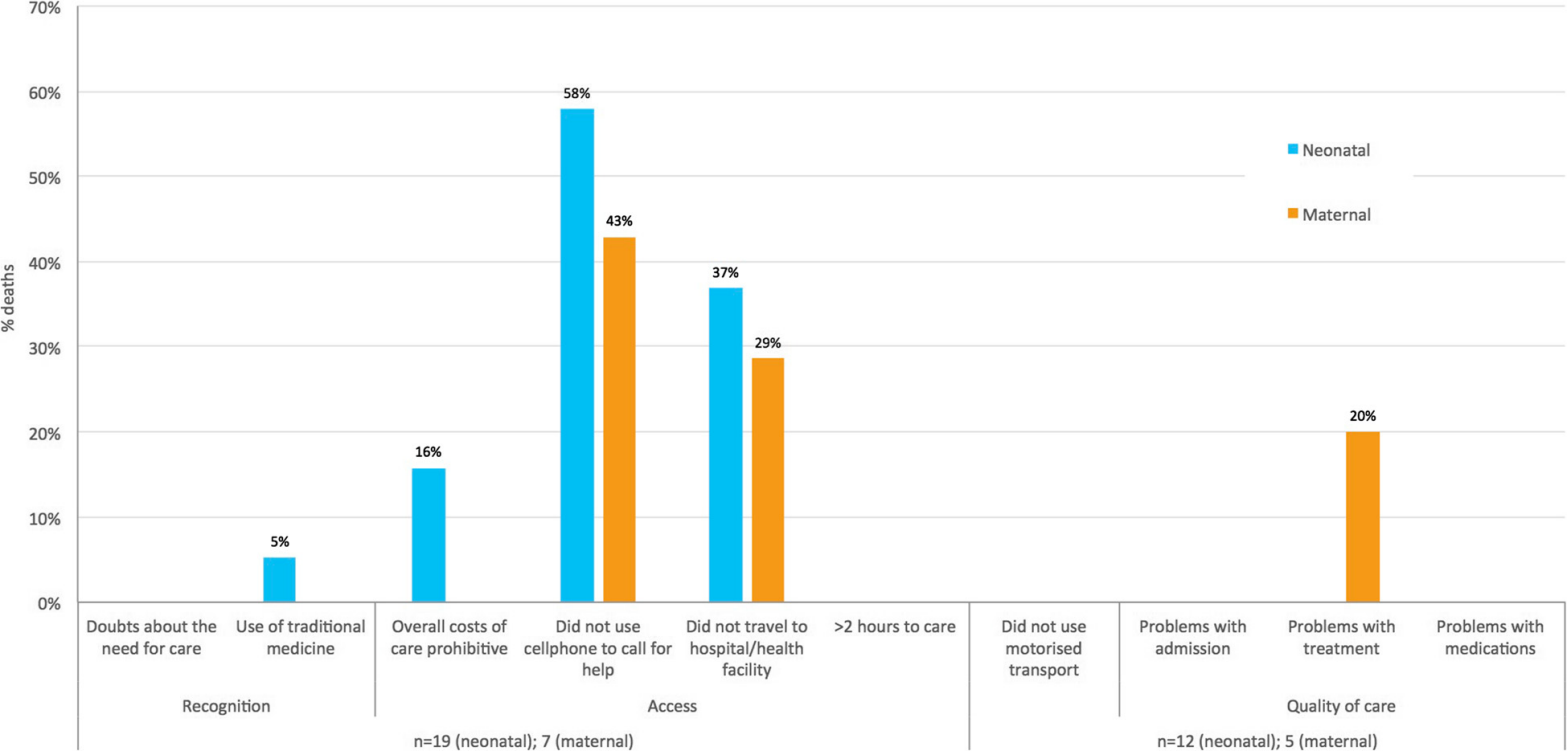
Frequencies of responses to new social and health systems indicators, neonatal and maternal deaths (*n* = 19 and 7)

A different pattern was observed for deaths owing to external, neonatal and maternal CODs, and for the deaths for which a COD could not be concluded. Most of these deaths had not called for help (82.6 % and 57.4 % of external and indeterminate CODs respectively). And the majority had not travelled to a facility at the time of death (82.6 % and 63.0 % external and indeterminate CODs respectively) (Fig. 5). For maternal, external, indeterminate and neonatal deaths, there were markedly lower problems with unaffordable care (0 %, 4.7 %, 11.1 % and 15.8 % respectively) and less use of traditional medicine at the time of death (0 %, 1.2 %, 7.4 %, and 5.3 % respectively). Of those who travelled to facilities at the time of death (15/86, 17.4 % external deaths; 20/54, 37.0 % indeterminate deaths; 12/19, 63.2 % neonatal deaths; and 5/7, 71.4 % maternal deaths) small proportions had problems with quality of care and the vast majority had used motorised transport (Figs. 5 and 6).

**Fig. 7:**
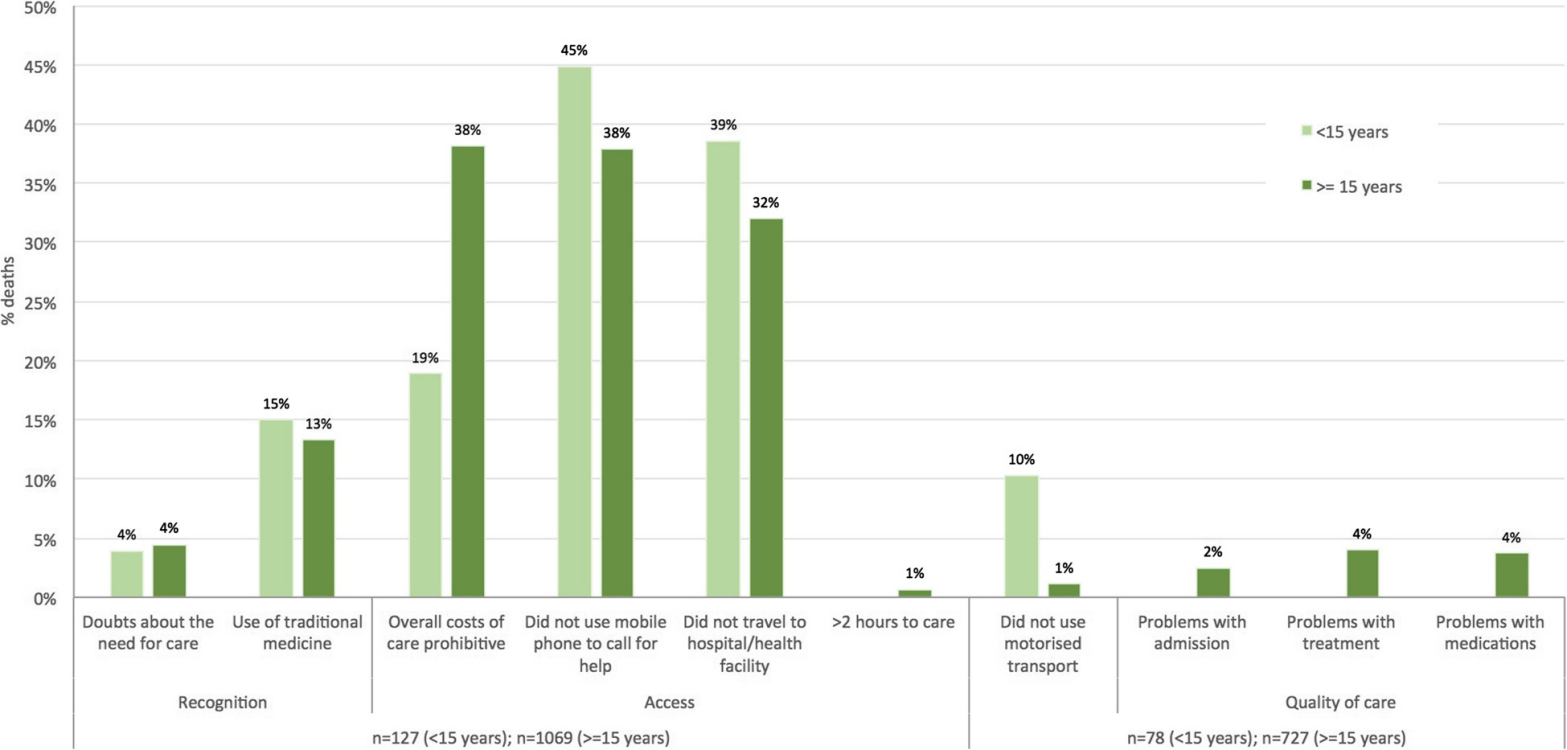
Frequencies of responses to new social and health systems indicators, <=14 years and > =15 years deaths (*n* = 127 and 1069)

The new indicators were also disaggregated by age and sex. Among men and women and for deaths >15 years, problems reported in the care pathway did not differ substantially and followed the general trend (i.e. 30-40 % reporting unaffordable costs, not calling for help, and not going to hospital). Whereas in the age groups of 15 years and less, there were fewer problems with costs but more with not calling for help or going to the hospital. In a similar sense to the trends described above, those that travelled to facilities (78/127, 61.4 % deaths less than 15 years; 727/1069, 68.0 % deaths more than or equal to 15 years) there were fewer problems with quality of services and using motorised transport (Table 4, Fig. 7).

**Table 4:** Absolute and relative frequencies of social and health systems indicators as proportions of numbers of deaths, by age groups

	Recognition		Access					Quality of care			
Age group	Doubts about the need for care	Use of traditional medicine	Overall costs prohibitive	Did not use cellphone	Did not travel to hospital/ health facility	>2 h to hospital/ health facility	Did not use motor transport^a^	Problems with admission^a^	Problems with treatment^a^	Problems with medications^a^	Total number of deaths *n* (%)
Neonate (<28 days)	2 (8.7)	3 (13.0)	3 (13.0)	13 (56.5)	13 (56.5)						23 (1.9)
Infant (1–11 months)		8 (24.2)	7 (21.2)	12 (36.4)	9 (27.3)		5 (20.8)				33 (2.8)
Under 5 (1–4 years)	3 (5.6)	8 (14.8)	10 (18.5)	25 (46.3)	20 (37.0)		5 (8.8)				54 (4.5)
Child (5–14 years)			4 (23.5)	7 (41.2)	7 (41.2)						17 (1.4)
Adult (15–49 years)	26 (4.8)	83 (15.5)	225 (41.9)	188 (35.0)	142 (26.4)	7 (1.3)	4 (1.0)	9 (2.3)	17 (4.3)	16 (4.1)	537 (44.9)
Mid-age (50–64 years)	7 (4.1)	18 (10.5)	61 (35.5)	69 (40.1)	49 (28.5)		2 (1.6)	4 (3.3)	5 (4.1)	10 (8.1)	172 (14.4)
Elder (65-84+ years)	15 (4.2)	41 (11.4)	122 (33.9)	148 (41.1)	151 (41.9)		2 (1.0)	5 (2.4)	7 (3.3)	1 (0.5)	360 (30.1)
											1196 (100.0)
Total number of indicators reported *n* (%)	53 (4.4)	161 (13.5)	432 (36.1)	462 (38.6)	391 (32.7)	7 (0.6)	16 (2.0)	18 (2.2)	29 (3.6)	27 (3.4)	

^a^Denominator for the relative frequency was the number of deaths that had travelled to a hospital or health facility

N.B. Respondents were able to indicate more than one social and health system indicator for each death reported. Proportional frequencies of the new indicators therefore sum to >100 %

## Discussion

This paper presents a development to VA as the method transitions towards routine application. In this section, we make broad statements about the findings relative to the overall profile of burden of disease in South Africa and develop an illustrative set of interpretations of policy and planning in order to explore the practical utility of the method for further development.

The results suggest that in Agincourt, there is double burden of communicable and non-communicable conditions, with comparatively lower levels of external, neonatal and maternal mortality. This is characteristic of a middle-income country in epidemiological transition [36]. Complex and dynamic health priorities present particular challenges for health systems where large numbers also face social exclusion, especially in deeply unequal societies where critical limiting factors arising from social and health system contexts have important roles in survival.

The new indicators were suggestive of multiple problems with access to services at the time of death. Over a third of deaths did not travel to a facility at the time of death, did not call for help, and found the overall costs of care unaffordable. This pattern was observed consistently across age and sex sub-groups. These issues clearly relate to and reinforce one another: if care is unaffordable then people are unlikely to call for help or travel to facilities at the time of death. Considering health systems as core social institutions, the exclusion of those unable to meet the resource requirements of the acute situation may become normalised through repeated claims for care that are unaffordable and unsuccessful [13].

Markedly higher proportions of deaths owing to external, neonatal and maternal causes did not travel to a facility or call for help, but had fewer problems with costs. An overall acute/chronic distinction explains this difference. Deaths in pregnancy, among children and due to accidents or assaults have unexpected and rapid onsets which may make calling for help and getting to a hospital difficult, but are less likely to have problems with costs. Patients with chronic illnesses by comparison are likely to have many more presentations for care in the management of long-term conditions, and so may experience more severe shocks from costs and problems across the care pathway. Assuming this pattern is valid, there may be reason to suspect that the 116 deaths for which no CODs were concluded were due to conditions with acute onset. In this sense, the new indicators also have the potential to inform medical interpretations and COD conclusions in VA.

### Methodological reflections

The results are the first analysis of the new social and health systems indicators in the WHO SF-VA and should be considered preliminary and with the following limitations in mind. Firstly, although fewer problems were reported with quality of care and recognition of severity, this does not necessarily indicate their absence. People who died outside facilities are less likely to have had problems with quality of care, regardless of whether problems exist. Indeed serious problems with quality of services and widespread traditional beliefs have been documented in Agincourt, albeit in research on general rather than on end of life care [23, 24]. Additionally, respondents may report on issues of access more than with quality of care given that they directly experienced the former to a greater extent. The results on quality of care should therefore be viewed with some caution and require further investigation [14].

Despite some potential sources of bias, the SF-VA identified patterns of problems in care seeking and utilisation that varied by categories of medical cause. This information on background characteristics of deaths investigated in VA is not available from other sources. The indicators provide a 100 % consistent and reproducible means to gain information on social and health systems determinants of potentially unregistered and uncounted deaths. This is a relatively unexamined aspect in investigations of how and why people die, particularly in the context of routine monitoring.

### Future directions

VA is a method to investigate deaths that occur without registration and/or outside facilities, which to date has been used in research studies and health and demographic surveillance, and which is currently in transition towards application on a wider scale as part of CRVS systems. Integrating the system into standard VA interpretation and mortality classification systems is therefore a natural next step to promote the recording and interpretation of information on critical limiting factors that arise form social and health systems contexts. The existing system that InterVA corresponds to consists of more than 68,000 codes for physiological states, processes and circumstances surrounding injury (Lancet. ICD-10: there’s a code for that. Editorial. [32]). Despite comprehensive coverage of ‘proximate determinants’, the system does not currently record much information on intermediate and distal or social and health systems determinants. ICD does consider contributory factors as: ‘the conditions that exist prior to the development of the underlying cause, or that develop during’ [48:734], and maternal death classifications were recently extended to include the cause categories related to ‘unanticipated complications of management’ [44]. The wording is suggestive of the potentially punitive implications for providers however, a further issue to take forward in future.

The information can also be augmented through stakeholder consultations with the public and health systems practitioners. Embedding VA within a broader community-led process examining the method, results and implications for local service planning could confer validity gains, generating more meaningful data for local decisions regarding service organisation and delivery [33, 35]. Practitioner and planner evaluations of VA can also provide a means to foster closer collaboration between research and policy, encouraging collective ownership between those who produce and use evidence as part of a broader health policy and systems research approach [6, 30, 56]. On this basis, the methods and results are currently being contextualised with communities and local health authorities in Mpumalanga to further develop recommendations for services and the data interpretation sequence. A brief illustrative health systems and policy interpretation is provided below to inform reflections on the utility of the method for local (district and provincial level) health planning.

### Substantive interpretation

The new indicators were suggestive of multiple problems with access to services at the time of death. The consistency of the trend suggests that actions to address these issues may have the potential to improve care and outcomes for a range of conditions. In terms of transport barriers, Mpumalanga province operates 12 ambulances for obstetric emergencies, and a toll-free helpline for emergency medical services [41]. Despite this, 43 % of the maternal deaths investigated did not call for help using a cellphone and 29 % did not travel to a facility at the time of death. Given the majority of those who did make the journey to a facility used motorised transport, the results suggests that informing decision-making for individuals, families and communities to seek care in obstetric emergencies may be beneficial. Furthermore, given that one third of all deaths did not travel to a facility, extending transport interventions may be a further priority locally.

In terms of affordability of care, although all state services are chargeable in Mpumalanga, (with the exception of Primary Health Care [PHC]), the provincial hospital fees manual states that no patient is required to meet all costs should they incur excessive financial burden, and that people with disabilities, recipients of social grants or formally unemployed may also qualify for fully subsidised health care [45]. Despite these provisions, prohibitive costs were reported consistently. This may be linked to the impact of indirect costs of care (transport costs, medications, food etc.), in combination with direct costs of services where they apply. In this sense, the implementation of National Health Insurance system launched in 2012/13 is relevant as a major commitment to equitable and affordable access for the population [15]. Extending VA to provide information on social and health systems determinants of deaths could provide important information on the effects of the policy in terms of health outcomes and key care processes at household level in future applications.

Despite provisions to reduce financial and transport barriers to access, the results suggest that large numbers of people face serious and multiple issues with access to services at and around the time of death. This suggests that community education fora to provide people with information on health care provisions and entitlements may be beneficial. A PHC re-engineering policy was introduced in 2011 to formalise and expand the roles of Community Health Workers (CHWs) through Ward-Based Outreach Teams (WBOTs), strengthen services in schools, and provide specialist teams for maternal and child health [16]. A further health systems interpretation is therefore to develop the relationship between WBOTs, the health authority and the community to improve connections and exchanges of information between health authorities and communities.

## Conclusions

Mortality that occurs outside health and/or civil registration systems constitutes the health of disadvantaged people. To build more complete renditions of, and thus responses to, complex and socially determined burdens of disease it is necessary to consider the social and health systems contexts in which health conditions are situated. This paper describes an extension to the standard VA interview to collect new information on social and health systems determinants of deaths. We sought to collect data not available from other sources to facilitate public health interpretations of deaths. The purpose relates to the transition of VA from a research to a service environment.

The analysis demonstrates the utility of collecting standard information on the circumstances, events and critical limiting factors that arise from local contexts. Through a simple descriptive analysis, it was possible to identify multiple barriers to access in end of life care, which collectively may be insurmountable for many. The consistency of the trend also suggests that actions to address these issues, by strengthening and promoting existing provisions to address financial and barriers to access, may have the potential to make positive impacts across a range of conditions.

Supplementing VA with questions on social and health system circumstances provides complementary information to CSMFs with a practical utility for service organisation and delivery. The data can be further augmented through collaborative analysis and interpretation by health authorities and communities. In this sense, VA can be considered as a basis from which to develop co-constructed practical knowledge built from multiple perspectives and embedded in local policy context, as a move towards more plural and people-centred health systems research.

## Abbreviations

CHW, community health worker; COD, cause of death; CSMF, cause-specific mortality fraction; CVD, cardiovascular disease; HDSS, health and demographic surveillance system; HIV/AIDS, human immunodeficiency virus/acquired immunodeficiency syndrome; ICD, International classification of diseases; INDEPTH, an International Network for the Demographic Evaluation of Populations and their health; InterVA, interpreting verbal autopsy; LMIC, low and middle-income country; PHC, primary health care; RTA, road traffic accident; SA, social autopsy; SF-VA, short-form verbal autopsy; VA, verbal autopsy; WBOT, ward-based outreach teams; WHO, World Health Organization

## Additional files

Additional file 1: Table S1. WHO VA treatment and health service use final illness [54]. Elements of the health care interaction examined in social autopsy studies [31]. Treatment and health care service use for the final illness module INDEPTH Iganga/Mayuge Verbal and Social Autopsy Instrument [4]. (DOC 120 kb)

Additional file 2: Table S2. Cause-specific mortality fraction (CSMF): all deaths and age/sex sub-groups. (DOC 99 kb)

Additional file 3: Table S3. Cause of death categories. (DOC 94 kb)

Additional file 4: Table S4. Social and health systems indicators by COD and COD categories. Social and health systems indicators by age and sex sub-groups. (DOC 192 kb)
