# Erratum to: Moving from medical to health systems classifications of deaths: extending verbal autopsy to collect information on the circumstances of mortality

**DOI:** 10.1186/s41256-016-0008-5

**Published:** 2016-07-19

**Authors:** Lucia D’Ambruoso, Kathleen Kahn, Ryan G. Wagner, Rhian Twine, Barry Spies, Maria van der Merwe, F. Xavier Gómez-Olivé, Stephen Tollman, Peter Byass

**Affiliations:** 1 Institute of Applied Health Sciences, University of Aberdeen, Scotland, UK; 2 Umeå Centre for Global Health Research, Umeå University, Umeå, Sweden; 3 MRC/Wits Rural Public Health and Health Transitions Research Unit, School of Public Health, Faculty of Health Sciences, University of the Witwatersrand, Johannesburg, South Africa; 4 Directorate for Maternal, Child, Women and Youth Health and Nutrition, Mpumalanga Department of Health, Nelspruit, Mpumalanga South Africa; 5 INDEPTH: An International Network for the Demographic Evaluation of Populations and Their Health, Accra, Ghana

## Erratum

After publication of the original article [1] it was brought to our attention that a couple of typos were incorporated into the published text. Please note that the third author name was incorrectly written as ‘Ryan G. Wager’. The correct author name should be ‘Ryan G. Wagner’.

Secondly, there was a repetition of the word ‘deaths’ in the first sentence of the results paragraph.

These have both now been corrected on the BioMed Central website.
