# Supplementary material for: Moving from medical to health systems classifications of deaths: extending verbal autopsy to collect information on the circumstances of mortality
Source: Glob Health Res Policy. 2016 Jun 15;1:2. doi: 10.1186/s41256-016-0002-y (PMC5675065; doi:10.1186/s41256-016-0002-y)
Supplement: Supplementary file 1 — WHO VA treatment and health service use final illness [54]. Elements of the health care interaction examined in social autopsy studies [31]. Treatment and health care service use for the final illness module INDEPTH Iganga/Mayuge Verbal and Social Autopsy Instrument [4]. (DOC 120 kb) [file 41256_2016_2_MOESM1_ESM.doc]

Supplementary Material 1.1: WHO VA Treatment and health service use for the final illness [8]

| DEATH OF A PERSON AGED 15 YEARS AND ABOVE | |
| --- | --- |
| 1001 | Did s/he receive any treatment for the illness that led to death? |
| 1002 | Can you please list the drugs s/he was given for the illness that led to death? |
| 1003 | What type of treatment did s/he receive:  1. Oral rehydration salts and/or IV fluids (drip) treatment?  2. Blood transfusion?  3. Treatment/food through a tube passed through the nose?  4. Any other treatment? |
| 1004 | Please tell me at which of the following places/facilities s/he received treatment during the illness that led to death:  1. Home?  2. Traditional healer?  3. Government clinic?  4. Government hospital?  5. Private clinic?  6. Private hospital?  7. Pharmacy / drug seller / store?  8. Any other place or facility? |
| 1005 | In the month before death, how many contacts with the formal health did s/he have? |
| 1006 | Did a healthcare worker tell you the cause of death? |
| 1007 | What did the health worker say? |
| 1008 | Did s/he have an operation for the illness? |
| 1009 | How long before death did s/he have the operation? |
| 1010 | On what part of the body was the operation? |

Supplementary Material 1.2: Elements of the healthcare interaction examined in social autopsy studies (adapted [21])

|  | |  | Element of health care utilisation | | | | | | | |  |  |  |
| --- | --- | --- | --- | --- | --- | --- | --- | --- | --- | --- | --- | --- | --- |
| Study/Setting | | Illness recognition | Home care | Recognition of severe illness | Times healthcare sought | Sequence of healthcare sought | Type of healthcare sought | Care seeking delays | Care seeking constraints | Quality of care | Referral | Compliance with home care / referral advice | Pathway to survival model |
| Sustrisna 1993 | Indonesia |  |  |  |  |  | x |  | x |  |  |  |  |
| Gutierrez 1994 | Mexico |  |  |  |  |  | x | x |  | x | x |  |  |
| Sodermann 1997 | Guinea-Bissau |  |  |  | x |  | x | x |  | x |  |  |  |
| Aguilar 1998 | Bolivia | x | x | x |  | x | x | x |  | x |  | x | x |
| de Bocaletti 1999 | Guatemala | x | x | x |  |  | x | x | x | x |  | x | x |
| de Souza 2000 | Brazil |  | x | x | x | x | x | x | x | x |  | x | x |
| RACHA 2000 | Cambodia | x | x | x | x | x | x | x | x | x |  | x | x |
| Bhandari 2002 | India |  |  | x | x | x | x | x |  | x | x | x | x |
| Schumacher 2002 | Guinea | x | x | x | x | x | x | x | x | x |  | x | x |
| Hinderaker 2003 | Tanzania |  |  |  |  |  |  | x |  | x |  | x |  |
| de Savigny 2004 | Tanzania |  | x |  | x | x | x |  |  |  |  |  |  |
| Bojalil 2007 | Mexico |  |  | x | x |  | x | x | x | x |  |  | x |
| Beiersmann 2007 | Burkina Faso |  |  |  |  |  | x |  | x |  |  |  |  |
| Waiswa 2010 | Uganda |  |  | x |  |  |  | x |  | x |  |  | x |
| Fawcus 1996 | Zimbabwe |  |  |  |  |  |  | x | x | x | x | x |  |
| Castro 2000 | Mexico |  |  | x | x |  | x | x | x | x | x |  |  |
| Supratikto 2002 | Indonesia |  |  |  |  |  | x | x | x | x |  |  |  |
| Bartlett 2005 | Afghanistan |  |  |  |  |  | x | x | x | x |  |  |  |
| Campbell 2005 | Egypt |  |  | x |  |  | x | x |  | x |  |  |  |
| UNICEF 2010 | India |  |  | x | x | x | x | x | x |  |  |  |  |
| Jafaray 2009 | Pakistan |  |  | x |  |  | x | x | x | x | x |  |  |
| D'Ambruoso 2010 | Burkina Faso, Indonesia |  |  |  |  |  |  | x | x | x |  |  |  |
|  | N (%) | 4 (18) | 6 (27) | 12 (55) | 9 (41) | 7 (32) | 18 (82) | 19 (86) | 14 (64) | 18 (82) | 5 (23) | 8 (36) | 8 (36) |

Supplementary Material 1.3: Treatment and healthcare service use for the final illness module INDEPTH Iganga/Mayuge Verbal and Social Autopsy Instrument [20]

| ADULT DEATHS | |
| --- | --- |
| 10.1 | Symptoms in chronological order |
| 10.2 | How long after you recognized the first symptom did s/he die? |
| 10.3 | Did s/he receive any treatment for the illness that led to death? |
| 10.4 | Why did s/he not receive any treatment? |
| 10.5 | How was s/he treated at home? |
| 10.6 | Can you please list the drugs s/he was given for the illness that led to death? |
| 10.7 | What type of treatment was given? |
| 10.8 | How much time after illness started was care initialized at home? |
| 10.9 | Was s/he brought outside the home for care while s/he had this illness? |
| 10.10 | Why did you not take him/her for outside care? |
| 10.11 | On which day after the first symptom was s/he brought outside the home for care? |
| 10.12 | Where did you go to seek treatment for the final illness? |
| 10.13 | In the month before death, how many contacts with formal health services did s/he have? |
| 10.14 | Did s/he have any operation for the illness? |
| 10.14.1 | How long before death did s/he have the operation? |
| 10.14.2 | On what part of the body was the operation? |
| 10.15 | How much did you pay for transport during the final illness episode? |
| 10.16 | How much did you pay for treatment and other costs related to the care of the baby (incl. fees for admission, consultation, lab tests, and consumables etc.)? |
| 10.17 | How much did you pay for other costs (incl. accommodation, feeding etc.)? |
|  |  |
| CHILD DEATHS | |
| 9.1 | Symptoms in chronological order (after illness started) |
| 9.2 | When you first noticed that s/he was ill, was s/he [feeding / alter / active] |
| 9.3 | Did the child receive any treatment before s/he died? |
| 9.4 | Why did the child not receive any treatment? |
| 9.5 | How was s/he treated at home? (Before child was taken to an outside provider) |
| 9.6 | What type of treatment was given to the child in the home? |
| 9.7 | For how many days did you give each treatment? |
| 9.8 | How much time after illness started was care initialized at home? |
| 9.9 | Who decided that giving the home care was the right thing to do at the time? |
| 9.10 | Was the child brought outside the home for care while s/he had the illness? |
| 9.11 | Why did you not take the child for outside care? |
| 9.12 | Who decided not to take the child for outside care? |
| 9.13 | On which day after the first symptom was the child brought outside the home for care? |
| 9.14 | Where did you go to seek treatment? |
| 9.15 | Care sought in chronological order |
| 9.16 | When you first noticed that s/he was ill, how was his/her [feeding / alertness / activeness]? |
| 9.17 | How did you take the child to the first and last care provider? |
| 9.18 | After deciding to seek outside care, how much time passed? |
| 9.19 | What was the reason for delay? |
| 9.20 | How much time did it take to go to the first and last provider? |
| 9.21 | What kind of treatment was given to the child at the first and last care provider? |
| 9.22 | What type of treatment was given to the child? |
| 9.23 | For how many days did you give each treatment? |
| 9.24 | How long after you arrived at the care provider was treatment obtained? |
| 9.25 | How long was the child at the provider? |
| 9.26 | What did the provider suggest you do for the child's illness after leaving? |
| 9.27 | Were you able to follow all the advice? |
| 9.28 | Why were you not able to follow all the advice? |
| 9.29 | Was the child given any other antibiotic or antimalarial during the course of the illness? |
| 9.30 | Did you have to pay for the treatment that was given to you? |
| 9.31 | Was the child ever hospitalized during the final illness? |
| 9.32 | Was the child ever referred to another place of care during the final illness? |
| 9.33 | Where did the provider tell you take the child for referral? |
| 9.34 | What was the reason for the referral? |
| 9.35 | Was the child taken to the place where s/he was referred? |
| 9.36 | Why did you not take the child for referral care? |
| 9.37 | How much did you pay for transport during the final illness episode? |
| 9.38 | How much did you pay for other costs (incl. accommodation, feeding etc.)? |
| 9.39 | How much did you pay for treatment and other costs related to care of the baby (incl. fees for admission, consultation, lab tests, and consumables etc.)? |
| 9.40 | Did the child usually sleep under a bed net? |
