# Supplementary material for: Moving from medical to health systems classifications of deaths: extending verbal autopsy to collect information on the circumstances of mortality
Source: Glob Health Res Policy. 2016 Jun 15;1:2. doi: 10.1186/s41256-016-0002-y (PMC5675065; doi:10.1186/s41256-016-0002-y)
Supplement: Supplementary file 2 — Cause-specific mortality fraction (CSMF): all deaths and age/sex sub-groups. (DOC 99 kb) [file 41256_2016_2_MOESM2_ESM.doc]

Supplementary Material 2: Cause-specific mortality fraction (CSMF): all deaths, age/sex sub-groups

|  | Age groups | | | | | | | Sex | |  |
| --- | --- | --- | --- | --- | --- | --- | --- | --- | --- | --- |
| Cause of death (COD) | Neonate  (<28 d) | Infant  (1-11 m) | Under 5  (1-4 y) | Child  (5-14 y) | Adult  (15-49 y) | Mid-age  (50-64 y) | Elder  (65-84+ y) | Female | Male | n (%) |
| Acute respiratory infection including pneumonia |  | 9 | 11 | 7 | 84 | 26 | 35 | 86 | 86 | 172 (14.4) |
| HIV/AIDS related death |  | 2 | 14 | 1 | 120 | 21 | 13 | 106 | 65 | 171 (14.3) |
| Pulmonary tuberculosis |  |  | 1 |  | 94 | 22 | 37 | 58 | 96 | 154 (12.9) |
| Other and unspecified cardiac disease |  |  |  |  | 17 | 9 | 60 | 55 | 31 | 86 (7.2) |
| Asthma |  |  | 2 | 1 | 30 | 19 | 32 | 44 | 40 | 84 (7.0) |
| Stroke |  |  |  |  | 11 | 5 | 51 | 51 | 16 | 67 (5.6) |
| Indeterminate | 7 |  | 2 | 2 | 26 | 5 | 12 | 34 | 20 | 54 (4.5) |
| Respiratory neoplasms |  |  |  |  | 14 | 9 | 18 | 19 | 22 | 41 (3.4) |
| Acute abdomen |  | 1 | 1 |  | 11 | 6 | 19 | 21 | 17 | 38 (3.2) |
| Road traffic accident |  |  | 2 |  | 27 | 2 |  | 5 | 26 | 31 (2.6) |
| Malaria |  | 6 | 8 | 2 | 7 | 3 | 3 | 15 | 14 | 29 (2.4) |
| Acute cardiac disease |  |  |  |  | 6 | 8 | 13 | 13 | 14 | 27 (2.3) |
| Digestive neoplasms |  |  |  |  | 8 | 8 | 5 | 7 | 14 | 21 (1.8) |
| Intentional self-harm |  |  |  | 1 | 13 | 5 | 2 | 5 | 16 | 21 (1.8) |
| Diarrhoeal diseases |  | 8 | 5 |  | 2 | 1 | 4 | 13 | 7 | 20 (1.7) |
| Assault |  |  | 1 |  | 12 | 1 | 2 | 3 | 13 | 16 (1.3) |
| Chronic obstructive pulmonary disease |  |  |  |  | 3 | 5 | 7 | 5 | 10 | 15 (1.3) |
| Reproductive neoplasms male/female |  |  |  |  | 7 | 3 | 4 | 11 | 3 | 14 (1.2) |
| Breast neoplasms |  |  |  |  | 11 |  | 1 | 12 |  | 12 (1.0) |
| Diabetes mellitus |  |  |  |  | 3 | 1 | 8 | 6 | 6 | 12 (1.0) |
| Other and unspecified neoplasms |  |  |  |  | 3 | 3 | 4 | 4 | 6 | 10 (0.8) |
| Liver cirrhosis |  |  |  |  | 1 | 1 | 8 | 5 | 5 | 10 (0.8) |
| Meningitis and encephalitis |  | 1 | 1 |  | 5 | 1 |  | 6 | 2 | 8 (0.7) |
| Severe anaemia |  |  | 1 |  | 1 | 2 | 4 | 2 | 6 | 8 (0.7) |
| Renal failure |  |  |  |  | 2 | 3 | 3 | 1 | 7 | 8 (0.7) |
| Other and unspecified infectious disease |  | 1 |  | 1 | 2 |  | 3 | 1 | 6 | 7 (0.6) |
| Neonatal pneumonia | 7 |  |  |  |  |  |  | 6 | 1 | 7 (0.6) |
| Other transport accident |  |  |  | 1 | 4 | 1 |  |  | 6 | 6 (0.5) |
| Accidental drowning and submersion |  |  | 3 | 1 | 2 |  |  | 3 | 3 | 6 (0.5) |
| Other and unspecified NCD |  |  |  |  |  |  | 6 | 5 | 1 | 6 (0.5) |
| Severe malnutrition |  |  | 1 |  |  |  | 4 | 5 |  | 5 (0.4) |
| Congenital malformation | 1 | 2 | 1 |  |  |  |  | 1 | 3 | 4 (0.3) |
| Epilepsy |  | 1 |  |  | 1 |  | 1 | 2 | 1 | 3 (0.3) |
| Prematurity | 3 |  |  |  |  |  |  | 1 | 2 | 3 (0.3) |
| Birth asphyxia | 3 |  |  |  |  |  |  | 1 | 2 | 3 (0.3) |
| Other and unspecified external CoD |  | 2 |  |  | 1 |  |  |  | 3 | 3 (0.3) |
| Pregnancy-induced hypertension |  |  |  |  | 2 |  |  | 2 |  | 2 (0.2) |
| Obstetric haemorrhage |  |  |  |  | 2 |  |  | 2 |  | 2 (0.2) |
| Accidental exposure to smoke fire & flame |  |  |  |  |  | 2 |  |  | 2 | 2 (0.2) |
| Sepsis (non-obstetric) |  |  |  |  | 1 |  |  | 1 |  | 1 (0.1) |
| Oral neoplasms |  |  |  |  |  |  | 1 | 1 |  | 1 (0.1) |
| Abortion-related death |  |  |  |  | 1 |  |  | 1 |  | 1 (0.1) |
| Pregnancy-related sepsis |  |  |  |  | 1 |  |  | 1 |  | 1 (0.1) |
| Anaemia of pregnancy |  |  |  |  | 1 |  |  | 1 |  | 1 (0.1) |
| Neonatal sepsis | 1 |  |  |  |  |  |  |  | 1 | 1 (0.1) |
| Other and unspecified neonatal CoD | 1 |  |  |  |  |  |  |  | 1 | 1 (0.1) |
| Accidental fall |  |  |  |  | 1 |  |  |  | 1 | 1 (0.1) |
| Total, n | 23 | 33 | 54 | 17 | 537 | 172 | 360 | 621 | 575 | 1196 |
| % | 1.9 | 2.8 | 4.5 | 1.4 | 44.9 | 14.4 | 30.1 | 51.9 | 48.1 | 100.0 |
