# Supplementary material for: Moving from medical to health systems classifications of deaths: extending verbal autopsy to collect information on the circumstances of mortality
Source: Glob Health Res Policy. 2016 Jun 15;1:2. doi: 10.1186/s41256-016-0002-y (PMC5675065; doi:10.1186/s41256-016-0002-y)
Supplement: Supplementary file 3 — Cause of death categories. (DOC 94 kb) [file 41256_2016_2_MOESM3_ESM.doc]

Supplementary Material 3: COD Categories and medical CODs with ICD codes

| COD Category | Medical COD and ICD code |
| --- | --- |
| External | 12.01 Road traffic accident |
|  | 12.02 Other transport accident |
|  | 12.03 Accidental fall |
|  | 12.04 Accidental drowning and submersion |
|  | 12.05 Accidental expos to smoke fire & flame |
|  | 12.08 Intentional self-harm |
|  | 12.09 Assault |
|  | 12.99 Other and unspecified external CoD |
| indeterminate | 99 Indeterminate |
| Infectious | 01.01 Sepsis (non-obstetric) |
|  | 01.02 Acute respiratory infection including pneumonia |
|  | 01.03 HIV/AIDS related death |
|  | 01.04 Diarrhoeal diseases |
|  | 01.05 Malaria |
|  | 01.07 Meningitis and encephalitis |
|  | 01.09 Pulmonary tuberculosis |
|  | 01.99 Other and unspecified infect dis |
| Maternal | 09.02 Abortion-related death |
|  | 09.03 Pregnancy-induced hypertension |
|  | 09.04 Obstetric haemorrhage |
|  | 09.06 Pregnancy-related sepsis |
|  | 09.07 Anaemia of pregnancy |
| Neonatal | 10.01 Prematurity |
|  | 10.02 Birth asphyxia |
|  | 10.03 Neonatal pneumonia |
|  | 10.04 Neonatal sepsis |
|  | 10.06 Congenital malformation |
|  | 10.99 Other and unspecified neonatal CoD |
| Non-communicable | 02.01 Oral neoplasms |
|  | 02.02 Digestive neoplasms |
|  | 02.03 Respiratory neoplasms |
|  | 02.04 Breast neoplasms |
|  | 02.05 & 02.06 Reproductive neoplasms MF |
|  | 02.99 Other and unspecified neoplasms |
|  | 03.01 Severe anaemia |
|  | 03.02 Severe malnutrition |
|  | 03.03 Diabetes mellitus |
|  | 04.01 Acute cardiac disease |
|  | 04.02 Stroke |
|  | 04.99 Other and unspecified cardiac dis |
|  | 05.01 Chronic obstructive pulmonary dis |
|  | 05.02 Asthma |
|  | 06.01 Acute abdomen |
|  | 06.02 Liver cirrhosis |
|  | 07.01 Renal failure |
|  | 08.01 Epilepsy |
|  | 98 Other and unspecified NCD |

Supplementary Material 3: COD Categories and medical CODs with ICD codes

| COD Category | Medical COD and ICD code |
| --- | --- |
| External | 12.01 Road traffic accident |
|  | 12.02 Other transport accident |
|  | 12.03 Accidental fall |
|  | 12.04 Accidental drowning and submersion |
|  | 12.05 Accidental expos to smoke fire & flame |
|  | 12.08 Intentional self-harm |
|  | 12.09 Assault |
|  | 12.99 Other and unspecified external CoD |
| indeterminate | 99 Indeterminate |
| Infectious | 01.01 Sepsis (non-obstetric) |
|  | 01.02 Acute respiratory infection including pneumonia |
|  | 01.03 HIV/AIDS related death |
|  | 01.04 Diarrhoeal diseases |
|  | 01.05 Malaria |
|  | 01.07 Meningitis and encephalitis |
|  | 01.09 Pulmonary tuberculosis |
|  | 01.99 Other and unspecified infect dis |
| Maternal | 09.02 Abortion-related death |
|  | 09.03 Pregnancy-induced hypertension |
|  | 09.04 Obstetric haemorrhage |
|  | 09.06 Pregnancy-related sepsis |
|  | 09.07 Anaemia of pregnancy |
| Neonatal | 10.01 Prematurity |
|  | 10.02 Birth asphyxia |
|  | 10.03 Neonatal pneumonia |
|  | 10.04 Neonatal sepsis |
|  | 10.06 Congenital malformation |
|  | 10.99 Other and unspecified neonatal CoD |
| Non-communicable | 02.01 Oral neoplasms |
|  | 02.02 Digestive neoplasms |
|  | 02.03 Respiratory neoplasms |
|  | 02.04 Breast neoplasms |
|  | 02.05 & 02.06 Reproductive neoplasms MF |
|  | 02.99 Other and unspecified neoplasms |
|  | 03.01 Severe anaemia |
|  | 03.02 Severe malnutrition |
|  | 03.03 Diabetes mellitus |
|  | 04.01 Acute cardiac disease |
|  | 04.02 Stroke |
|  | 04.99 Other and unspecified cardiac dis |
|  | 05.01 Chronic obstructive pulmonary dis |
|  | 05.02 Asthma |
|  | 06.01 Acute abdomen |
|  | 06.02 Liver cirrhosis |
|  | 07.01 Renal failure |
|  | 08.01 Epilepsy |
|  | 98 Other and unspecified NCD |
