# Supplementary material for: Moving from medical to health systems classifications of deaths: extending verbal autopsy to collect information on the circumstances of mortality
Source: Glob Health Res Policy. 2016 Jun 15;1:2. doi: 10.1186/s41256-016-0002-y (PMC5675065; doi:10.1186/s41256-016-0002-y)
Supplement: Supplementary file 4 — Social and health systems indicators by COD and COD categories. Social and health systems indicators by age and sex sub-groups. (DOC 192 kb) [file 41256_2016_2_MOESM4_ESM.doc]

Supplementary Material 4.1: Social and health system indicators by COD and COD categories

|  | Recognition | | Access | | | | | Quality of care | | |  |
| --- | --- | --- | --- | --- | --- | --- | --- | --- | --- | --- | --- |
| Cause of death (COD)/COD Category | Doubts | Traditional medicine | Overall costs prohibitive | Non-use  cellphone | Did not travel to hospital/health facility | >2h to  hospital/health facility | Non-use  motor transport | Problems with admission | Problems with treatment | Problems with medications | No. deaths |
| *Infectious* | *28* | *102* | *254* | *178* | *205* | *6* | *132* | *8* | *17* | *19* | *562* |
| Acute respiratory infection incl. pneumonia | 5 | 21 | 52 | 49 | 49 | 1 | 38 | 2 | 3 | 4 | 172 |
| HIV/AIDS related death | 14 | 41 | 94 | 56 | 66 | 1 | 44 | 2 | 6 | 6 | 171 |
| Pulmonary tuberculosis | 7 | 24 | 92 | 53 | 68 | 3 | 24 | 4 | 7 | 5 | 154 |
| Malaria | 1 | 8 | 6 | 9 | 8 |  | 10 |  | 1 | 1 | 29 |
| Diarrhoeal diseases |  | 4 | 6 | 5 | 9 |  | 11 |  |  | 1 | 20 |
| Meningitis and encephalitis |  | 2 | 2 | 3 | 2 |  | 2 |  |  | 2 | 8 |
| Other and unspecified infect disease | 1 | 1 | 1 | 3 | 3 | 1 | 3 |  |  |  | 7 |
| Sepsis (non-obstetric) |  | 1 | 1 |  |  |  |  |  |  |  | 1 |
| *NCD* | *22* | *53* | *165* | *168* | *205* | *1* | *160* | *8* | *10* | *8* | *468* |
| Other and unspecified cardiac disease | 3 | 5 | 32 | 28 | 38 | 1 | 28 | 3 | 3 | 1 | 86 |
| Asthma | 5 | 10 | 12 | 27 | 29 |  | 28 |  | 3 |  | 84 |
| Stroke | 3 | 8 | 22 | 27 | 33 |  | 31 |  |  |  | 67 |
| Respiratory neoplasms | 1 | 6 | 21 | 10 | 13 |  | 7 |  |  | 1 | 41 |
| Acute abdomen | 1 | 9 | 13 | 10 | 12 |  | 10 | 1 |  | 1 | 38 |
| Acute cardiac disease | 1 | 1 | 3 | 16 | 19 |  | 18 |  |  |  | 27 |
| Digestive neoplasms | 1 | 2 | 14 | 11 | 7 |  | 5 |  |  | 2 | 21 |
| Chronic obstructive pulmonary disease |  | 1 | 5 | 5 | 10 |  | 4 | 1 | 1 |  | 15 |
| Reproductive neoplasms male/female | 2 | 5 | 8 | 3 | 6 |  | 3 | 2 | 2 | 2 | 14 |
| Breast neoplasms | 1 |  | 6 | 3 | 8 |  | 4 |  |  |  | 12 |
| Diabetes mellitus |  | 1 | 4 | 6 | 6 |  | 4 |  |  |  | 12 |
| Other and unspecified neoplasms | 1 | 1 | 8 | 5 | 3 |  | 3 |  |  |  | 10 |
| Liver cirrhosis |  | 2 | 7 | 1 | 5 |  | 4 |  | 1 |  | 10 |
| Severe anaemia |  |  | 2 | 3 | 4 |  | 1 |  |  | 1 | 8 |
| Renal failure |  |  | 2 | 5 | 4 |  | 4 |  |  |  | 8 |
| Other and unspecified NCD |  |  | 3 | 4 | 4 |  | 2 |  |  |  | 6 |
| Severe malnutrition | 2 | 1 | 1 | 3 | 3 |  | 3 |  |  |  | 5 |
| Epilepsy |  |  | 1 | 1 | 1 |  | 1 |  |  |  | 3 |
| Oral neoplasms | 1 | 1 | 1 |  |  |  |  | 1 |  |  | 1 |
| *External* |  | *1* | *4* | *71* | *71* |  | *72* |  |  |  | *86* |
| Road traffic accident |  |  |  | 26 | 24 |  | 24 |  |  |  | 31 |
| Intentional self-harm |  |  | 4 | 15 | 17 |  | 17 |  |  |  | 21 |
| Assault |  | 1 |  | 13 | 13 |  | 14 |  |  |  | 16 |
| Other transport accident |  |  |  | 6 | 6 |  | 6 |  |  |  | 6 |
| Accidental drowning and submersion |  |  |  | 6 | 6 |  | 6 |  |  |  | 6 |
| Other and unspecified external CoD |  |  |  | 3 | 3 |  | 3 |  |  |  | 3 |
| Accidental expos to smoke fire & flame |  |  |  | 2 | 2 |  | 2 |  |  |  | 2 |
| Accidental fall |  |  |  |  |  |  |  |  |  |  | 1 |
| *Indeterminate* | *3* | *4* | *6* | *31* | *35* |  | *34* | *2* | *1* |  | *54* |
| *Neonatal* |  | *1* | *3* | *11* | *12* |  | *7* |  |  |  | *19* |
| Neonatal pneumonia |  | 1 | 2 | 3 | 3 |  | 1 |  |  |  | 7 |
| Congenital malformation |  |  |  | 3 | 2 |  | 2 |  |  |  | 4 |
| Prematurity |  |  |  | 3 | 3 |  | 1 |  |  |  | 3 |
| Birth asphyxia |  |  |  | 1 | 2 |  | 2 |  |  |  | 3 |
| Neonatal sepsis |  |  |  | 1 | 1 |  | 1 |  |  |  | 1 |
| Other and unspecified neonatal CoD |  |  | 1 |  | 1 |  |  |  |  |  | 1 |
| *Maternal* |  |  |  | *3* | *4* |  | *2* |  | *1* |  | *7* |
| Pregnancy-induced hypertension |  |  |  | 2 | 2 |  | 1 |  |  |  | 2 |
| Obstetric haemorrhage |  |  |  |  |  |  |  |  | 1 |  | 2 |
| Abortion-related death |  |  |  | 1 | 1 |  |  |  |  |  | 1 |
| Pregnancy-related sepsis |  |  |  |  |  |  |  |  |  |  | 1 |
| Anaemia of pregnancy |  |  |  |  | 1 |  | 1 |  |  |  | 1 |
| Total, n | 53 | 161 | 432 | 462 | 391 | 7 | 16 | 18 | 29 | 27 | 1196 |
| % | 4.4 | 13.5 | 36.1 | 38.6 | 32.7 | 0.6 | 2.0 | 2.2 | 3.6 | 3.4 |  |

Supplementary Material 4.2: Social and health system indicators by age and sex sub-groups

|  | Recognition | | Access | | | | | Quality of care | | |  |
| --- | --- | --- | --- | --- | --- | --- | --- | --- | --- | --- | --- |
| Age/Sex | Doubts | Traditional medicine | Overall costs prohibitive | Non-use  cellphone | Did not travel to hospital/health facility | >2h to  hospital/health facility | Non-use  motor transport | Problems with admission | Problems with treatment | Problems with medications | No. deaths |
| *Female* | *31* | *83* | *236* | *218* | *272* | *2* | *200* | *9* | *16* | *17* | *621* |
| < 28 d | 2 | 2 | 2 | 8 | 11 |  | 8 |  |  |  | 14 |
| 1-11 m |  | 6 | 5 | 7 | 5 |  | 9 |  |  |  | 22 |
| 1-4 y | 2 | 2 | 6 | 8 | 11 |  | 7 |  |  |  | 23 |
| 5-9 y |  |  |  | 1 | 2 |  | 2 |  |  |  | 3 |
| 10-14 y |  |  | 2 |  |  |  |  |  |  |  | 2 |
| 15-19 y | 1 | 5 | 5 | 4 | 3 |  | 1 |  |  |  | 14 |
| 20-24 y | 2 | 6 | 12 | 5 | 8 |  | 8 | 1 | 2 | 1 | 25 |
| 25-29 y | 4 | 6 | 21 | 12 | 12 |  | 10 | 1 | 1 | 2 | 49 |
| 30-34 y | 1 | 6 | 25 | 18 | 22 |  | 12 |  | 2 | 4 | 58 |
| 35-39 y | 3 | 8 | 20 | 20 | 25 |  | 16 | 1 | 3 | 2 | 53 |
| 40-44 y | 4 | 8 | 28 | 19 | 17 | 1 | 9 | 1 | 1 |  | 50 |
| 45-49 y | 2 | 5 | 19 | 9 | 19 | 1 | 9 |  | 1 | 2 | 42 |
| 50-54 y | 1 | 2 | 14 | 10 | 11 |  | 5 | 2 | 2 | 3 | 27 |
| 55-59 y | 1 | 3 | 6 | 5 | 7 |  | 5 |  | 1 | 1 | 14 |
| 60-64 y |  | 2 | 5 | 7 | 10 |  | 7 | 1 | 2 | 1 | 22 |
| 65-69 y | 1 | 2 | 7 | 8 | 8 |  | 8 |  | 1 |  | 21 |
| 70-74 y |  | 3 | 8 | 8 | 6 |  | 4 | 1 |  |  | 23 |
| 75-80 y | 3 | 4 | 12 | 18 | 24 |  | 20 |  |  |  | 41 |
| 80-84 y | 1 | 5 | 19 | 21 | 31 |  | 21 |  |  | 1 | 53 |
| 85+ y | 3 | 8 | 20 | 30 | 40 |  | 39 | 1 |  |  | 65 |
| *Male* | *22* | *78* | *196* | *244* | *260* | *5* | *207* | *9* | *13* | *10* | *575* |
| < 28 d |  | 1 | 1 | 5 | 7 |  | 5 |  |  |  | 9 |
| 1-11 m |  | 2 | 2 | 5 | 5 |  | 5 |  |  |  | 11 |
| 1-4 y | 1 | 6 | 4 | 17 | 17 |  | 16 |  |  |  | 31 |
| 5-9 y |  |  |  | 4 | 3 |  | 3 |  |  |  | 6 |
| 10-14 y |  |  | 2 | 2 | 2 |  | 2 |  |  |  | 6 |
| 15-19 y | 1 | 2 | 2 | 2 | 2 |  | 2 |  |  |  | 6 |
| 20-24 y |  | 1 | 2 | 6 | 8 |  | 6 | 1 |  |  | 13 |
| 25-29 y | 2 | 6 | 10 | 23 | 20 |  | 20 |  | 1 |  | 37 |
| 30-34 y |  | 5 | 20 | 16 | 19 | 3 | 11 | 2 | 1 | 2 | 46 |
| 35-39 y | 4 | 9 | 24 | 23 | 28 | 1 | 19 |  | 2 | 1 | 56 |
| 40-44 y |  | 5 | 13 | 21 | 18 | 1 | 12 | 1 |  | 1 | 42 |
| 45-49 y | 2 | 11 | 24 | 10 | 19 |  | 11 | 1 | 3 | 1 | 46 |
| 50-54 y | 1 | 2 | 10 | 19 | 17 |  | 13 |  |  | 3 | 40 |
| 55-59 y | 3 | 5 | 9 | 8 | 7 |  | 7 |  |  | 1 | 25 |
| 60-64 y | 1 | 4 | 17 | 20 | 18 |  | 14 | 1 |  | 1 | 44 |
| 65-69 y | 2 | 3 | 11 | 10 | 10 |  | 7 |  |  |  | 28 |
| 70-74 y | 1 | 7 | 15 | 19 | 21 |  | 18 | 1 | 2 |  | 45 |
| 75-80 y | 1 | 2 | 12 | 9 | 12 |  | 10 | 1 | 2 |  | 24 |
| 80-84 y | 1 | 1 | 11 | 10 | 12 |  | 11 |  | 2 |  | 29 |
| 85+ y | 2 | 6 | 7 | 15 | 15 |  | 15 | 1 |  |  | 31 |
| Total, n | 53 | 161 | 432 | 462 | 391 | 7 | 16 | 18 | 29 | 27 | 1196 |
| % | 4.4 | 13.5 | 36.1 | 38.6 | 32.7 | 0.6 | 2.0 | 2.2 | 3.6 | 3.4 |  |
